# STK16 regulates actin dynamics to control Golgi organization and cell cycle

**DOI:** 10.1038/srep44607

**Published:** 2017-03-15

**Authors:** Juanjuan Liu, Xingxing Yang, Binhua Li, Junjun Wang, Wenchao Wang, Jing Liu, Qingsong Liu, Xin Zhang

**Affiliations:** 1 High Magnetic Field Laboratory, Chinese Academy of Sciences, Hefei, Anhui, 230031, P. R. China; 2 University of Science and Technology of China, Hefei, Anhui, 230036, P. R. China

## Abstract

STK16 is a ubiquitously expressed, myristoylated, and palmitoylated serine/threonine protein kinase with underexplored functions. Recently, it was shown to be involved in cell division but the mechanism remains unclear. Here we found that human STK16 localizes to the Golgi complex throughout the cell cycle and plays important roles in Golgi structure regulation. STK16 knockdown or kinase inhibition disrupts actin polymers and causes fragmented Golgi in cells. *In vitro* assays show that STK16 directly binds to actin and regulates actin dynamics in a concentration- and kinase activity-dependent way. In addition, STK16 knockdown or kinase inhibition not only delays mitotic entry and prolongs mitosis, but also causes prometaphase and cytokinesis arrest. Therefore, we revealed STK16 as a novel actin binding protein that resides in the Golgi, which regulates actin dynamics to control Golgi structure and participate in cell cycle progression.

The Golgi complex is a continuous membranous system that is localized to the perinuclear area in a cell. It has been shown that the Golgi complex plays essential roles in secretory trafficking, lipid biosynthesis, protein modifications and the sorting and transport of proteins^1^. In interphase mammalian cells, the Golgi apparatus consists of stacks of parallel aligned flattened membrane cisternae, which are further linked laterally by tubules to form a ribbon-like structure^2^. Current models suggest that the assembly of the Golgi ribbon is an actin- and microtubule- dependent process and that proper positioning and maintenance of the Golgi are required for polarized cellular trafficking and normal cell motility^3^,^4^,^5^,^6^,^7^,^8^,^9^,^10^,^11^. Microtubules (and microtubule-associated proteins) determine the localization of the Golgi ribbon around the centrosome; whereas the actin cytoskeleton maintains the continuity and flatness of cisternae in conjunction with other Golgi matrix proteins^5^.

Although how actin maintains the integrity of the Golgi complex structure remains to be further explored, it is likely that some Golgi resident proteins carry out their structural function via direct or indirect interaction with actin and/or actin associated proteins. Initially, actin toxins revealed putative involvement of the actin dynamics in Golgi structure maintenance. For example, F-actin depolymerization by Cytochalasin D (Cyto D) or Latrunculin B (Lat B) induces perforation/fragmentation and severe swelling of Golgi cisternae which leads to a complete cisternae disorganization^12^,^13^. In contrast, F-actin stabilization by Jasplakinolide produces large perforation/fragmentation but not cisternae swelling^12^, which indicates that a dynamic actin network plays essential roles in regulating the architecture of the Golgi complex. These morphological alterations may be due to hyperosmotic protein diffusion caused by actin transformation at the Golgi complex^12^,^14^. In addition, it is reported that interaction between GOLPH3 and myosin 18 A, an actin interacting protein, is required for extension of the Golgi ribbon and the formation of transport carriers^15^. Another recent example is that mammalian Mena, which directly enhances actin filament elongation by interacting with the barbed end of the actin filament, facilitates Golgi reassembly stacking protein 65 (GRASP65) oligomerization and promotes local actin polymerization to link Golgi stacks into a ribbon^16^. These studies indicate that a complex molecular machinery of actin regulators and its associated proteins regulate actin dynamics to control Golgi structure.

The complex organization of the Golgi ribbon is highly dynamic during cell division. The Golgi ribbon is cut into individual Golgi stacks in the G2 phase. Upon entry into mitosis, they are unstacked and undergo vesiculation until these fragments appear as the “Golgi haze” at metaphase^17^,^18^,^19^. This Golgi fragmentation is required not only for daughter cell inheritance, but also for mitotic entrance itself which is called the “Golgi mitotic checkpoint”. It involves multi-step Golgi fragmentation and releases some Golgi proteins that are important for mitosis^20^. Blocking the fragmentation process results in cell cycle arrest in G2. Further study identified early G2 as the specific stage of Golgi fragmentation^21^. GRASP-55/65, MEK1/ERK1c, and BARS have been identified to be relevant to the severing of the ribbon and recruitment/activation of proteins essential for entry into mitosis^22^,^23^, but the other mechanisms or proteins coordinating with them remains a matter for future investigations.

STK16 (serine/threonine kinase 16, also known as Krct, PKL12, MPSK1, and TSF-1), conserved among all of the eukaryotes, appears to be the first mammalian member of a new Ser/Thr kinase subfamily^24^,^25^,^26^,^27^. Previous studies, including ours, found that purified STK16 is able to phosphorylate 4EBP1 and DRG1, as well as autophosphorylation^28^,^29^. Although it has been discovered for around twenty years, the biological functions of STK16 are still not well understood. STK16 is a myristoylated and palmitoylated kinase, localizing to the Golgi and is believed to be involved in the regulation of sorting secretory soluble cargo into the constitutive secretory pathway at the trans-Golgi network^24^,^30^. Moreover, our previous study showed that STK16 depletion or kinase inhibition induced binucleated cells as well as cell accumulation in the G2/M-phase^29^. However, the molecular mechanism remains unknown. Here, we further explore the mechanism of STK16 by a combination of RNAi knockdown and STK16 specific inhibitor to reveal that STK16 directly binds and regulates actin dynamics to control the Golgi complex and cell cycle.

## Results

### STK16 localizes to the Golgi complex and STK16 depletion impairs Golgi complex integrity

**Figure 1:**
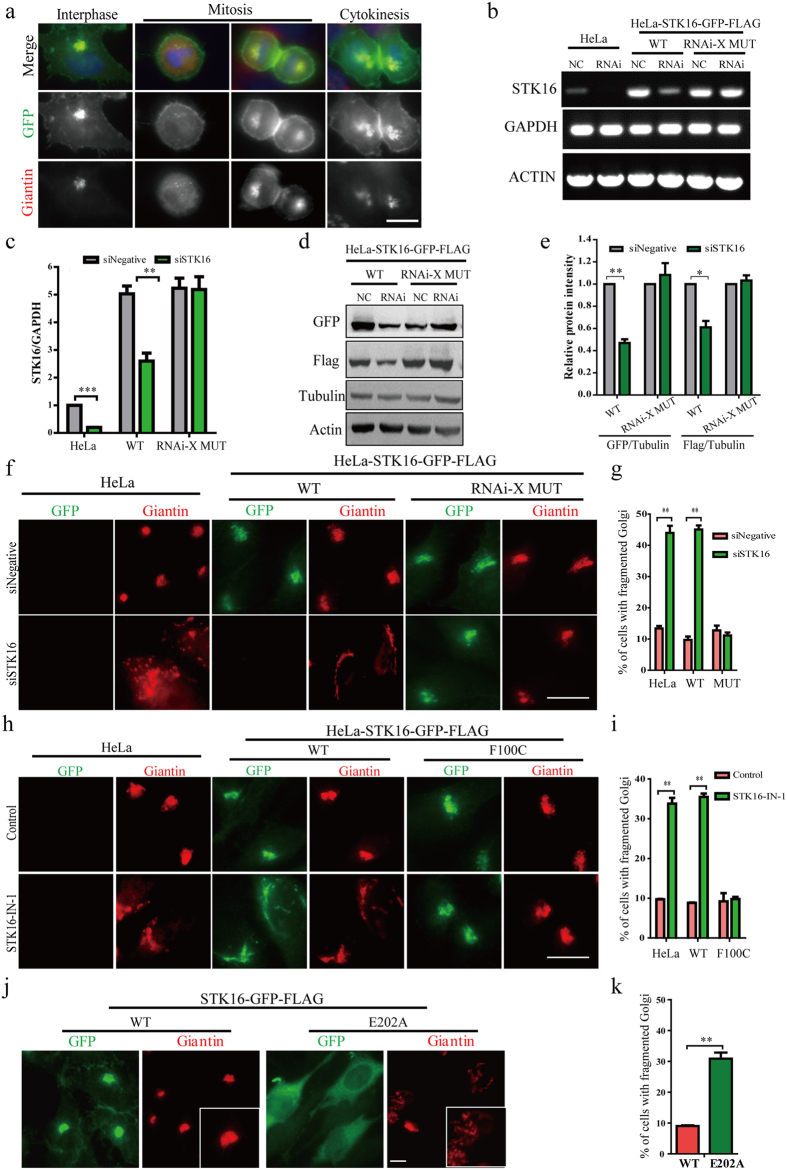
STK16 localizes to the Golgi complex and its depletion or kinase inhibition impairs Golgi integrity. (**a**) STK16 localizes to the Golgi complex during the whole cell cycle. HeLa-STK16-GFP-FLAG overexpression cell line grown on coverslips was fixed and stained for GFP (green) and Giantin (red). (**b–e**) STK16 RNAi specifically knockdown STK16. HeLa, and HeLa cells stably transfected with STK16-GFP-FLAG-WT or STK16-GFP-FLAG-MUT (RNAi resistant mutant) were transfected with control or STK16 siRNAs for 72 hours before they were lysed for RT-PCR (**b,c**) and western blots (**d,e**). (**f,g**) STK16 depletion disrupts Golgi complex integrity. HeLa cells and HeLa cells stably transfected with STK16-GFP-FLAG-WT or STK16-GFP-FLAG-MUT (RNAi resistant mutant) were transfected with control or STK16 siRNAs for 72 hours before they were harvested for immunofluorescence. Cells were fixed and stained with anti-GFP and anti-Giantin antibodies. Representative immunofluorescence images are shown in (**f**) and quantification results are shown in (**g**). (**h**) The inhibitor resistant mutant (F100C) rescues STK16-IN-1-induced Golgi structure disruption. HeLa and HeLa cells stably transfected with STK16-GFP-FLAG-WT or STK16-GFP-FLAG-F100C (drug resistant) were treated with 10 μM STK16-IN-1 for 6 hours. Cells were fixed and stained with GFP (green) and Giantin (red). (**i**) Quantification of the percentage of cells with fragmented Golgi complex in (**h**). (**j**) E202A, the kinase-dead mutant of STK16, induces fragmented Golgi. HeLa cells were transfected with STK16-GFP-FLAG-WT or kinase-dead mutant E202A for 48 hours before they were harvested for immunofluorescence using anti-GFP (green) and anti-Giantin (red) antibodies. (**k**) Quantification of % of cells with fragmented Golgi in (**j**) are shown. Experiments were repeated three times and representative images are shown. The data were analyzed using a student’s *t*- test (*p < 0.05, **p < 0.01, ***p < 0.001). Data show mean ± SEM, n = 3. Scale bar, 10 μm.

It was reported that STK16 protein is a Golgi-resident enzyme in murine and human cells^30^,^31^, but its function was not clear, especially for the human homologue. We first tried the anti-STK16 antibody from Sigma in the western blots and found that it gave multiple unspecific bands while only weakly recognized the endogenous STK16 (Supplementary Fig. S1a). In addition, this anti-STK16 antibody did not show any Golgi staining in HeLa, NIH-3T3, or MCF-7 cells as previous researches have shown^30^,^32^ (Supplementary Fig. S1b). Since it has been reported that His or GST tag has no influence on the kinase activity of STK16^26^, we constructed an expression vector containing the human STK16 cDNA with GFP and FLAG tag fused at the C-terminus of STK16 (STK16-GFP-FLAG) and transfected it into HeLa cells to establish a HeLa-STK16-GFP-FLAG-WT (wild-type) stable cell line. We found STK16-GFP-FLAG was enriched on the Golgi complex (Fig. 1a), which confirmed the results from the Antonio Bernad Group^30^,^31^. In contrast, the anti-STK16 antibody failed to stain the overexpressed STK16, which confirmed that this antibody is not applicable here (Supplementary Fig. S1c). Therefore, we chose to use the stable cell line and the anti-Flag, or anti-GFP antibodies, in Western Blot and immunofluorescence experiments for the rest of this study. We found that STK16 was associated with the Golgi structure throughout the whole cell cycle (Fig. 1a).

To unveil the function of STK16, we performed the RNAi knockdown experiment. In addition, to avoid potential off-target effects of RNAi and validate the RNAi knockdown specificity, we constructed a stable cell line with an RNAi resistant mutant (HeLa-STK16-GFP-FLAG-MUT), which contained silent mutations at the siRNA region so that it still expressed wild-type STK16 protein but escaped siRNA knockdown effect. Since the commercially available human STK16 antibody we purchased did not work well in our western blot or immunofluorescence experiments, we used RT-PCR to examine the STK16 levels in cells (Fig. 1b). As expected, the STK16 RNAi did knockdown STK16 levels in both HeLa and HeLa-STK16-GFP-FLAG-WT cells, but not in the RNAi-resistant mutant cells (Fig. 1b and c). Furthermore, the STK16 protein level was reduced by around 50 and 45% as measured by GFP and Flag antibodies (p < 0.01) in HeLa-STK16-GFP-FLAG-WT cells, but not in the RNAi-resistant mutant HeLa-STK16-GFP-FLAG-MUT cells (Fig. 1d and e). These results demonstrated the efficiency and specificity of STK16 RNAi.

We then investigated the function of STK16 on Golgi complex organization by RNAi depletion of STK16 in HeLa, HeLa-STK16-GFP-FLAG-WT, and HeLa-STK16-GFP-FLAG-MUT cells. Immunofluorescence experiments using Giantin as a Golgi marker showed that the knockdown of STK16 in HeLa and HeLa-STK16-GFP-FLAG-WT cells caused Golgi fragmentation with Golgi complex elements detaching from each other and dispersing into the cytoplasm (Fig. 1f). Quantification showed that STK16 knockdown increased the percentage of cells with fragmented Golgi complex from 13.4% to 43.9% in HeLa cells and from 9.7% to 45.0% in HeLa-STK16-GFP-FLAG-WT cells. However, HeLa-STK16-GFP-FLAG-MUT cells completely rescued the Golgi fragmentation caused by RNAi which confirmed the RNAi specificity (Fig. 1f and g). Thus, STK16 is required for maintaining Golgi complex integrity.

### The kinase activity of STK16 is critical for its regulation of Golgi complex integrity

In our previous study, we discovered a highly selective inhibitor against STK16 kinase, STK16-IN-1^29^. To examine whether the effect of STK16 on the Golgi complex is relevant to its kinase activity, we treated HeLa and HeLa-STK16-GFP-FLAG-WT cells with 10 μM STK16-IN-1 for 6 hours and found that STK16-IN-1 also caused Golgi complex fragmentation (Fig. 1h), which is similar to STK16 RNAi. To ensure that the STK16-IN-1 induced Golgi phenotype was specific, we used a HeLa-STK16-GFP-FLAG-F100C cell line (Fig. 1h) which has the hinge binding site of the STK16-IN-1 mutated and was reported to be resistant to STK16-IN-1^29^. Our results show that STK16-IN-1 increased the percentage of cells with fragmented Golgi complex from 9.7% to 33.8% in HeLa cells and from 8.8% to 35.5% in HeLa-STK16-GFP-FLAG-WT cells, but not in HeLa-STK16-GFP-FLAG-F100C cells (Fig. 1i), which confirmed the on-target effect of the STK16-IN-1.

Moreover, to confirm the role of the kinase activity of STK16 in Golgi integrity regulation, we transiently transfected a kinase-dead mutant, STK16-GFP-FLAG-E202A^31^, into HeLa cells and found that it significantly increases the percentage of cells with fragmented Golgi (Fig. 1j and k). These results all indicated that the kinase activity is critical for STK16 to function in Golgi assembly.

### STK16 functions in Golgi reassembly

**Figure 2:**
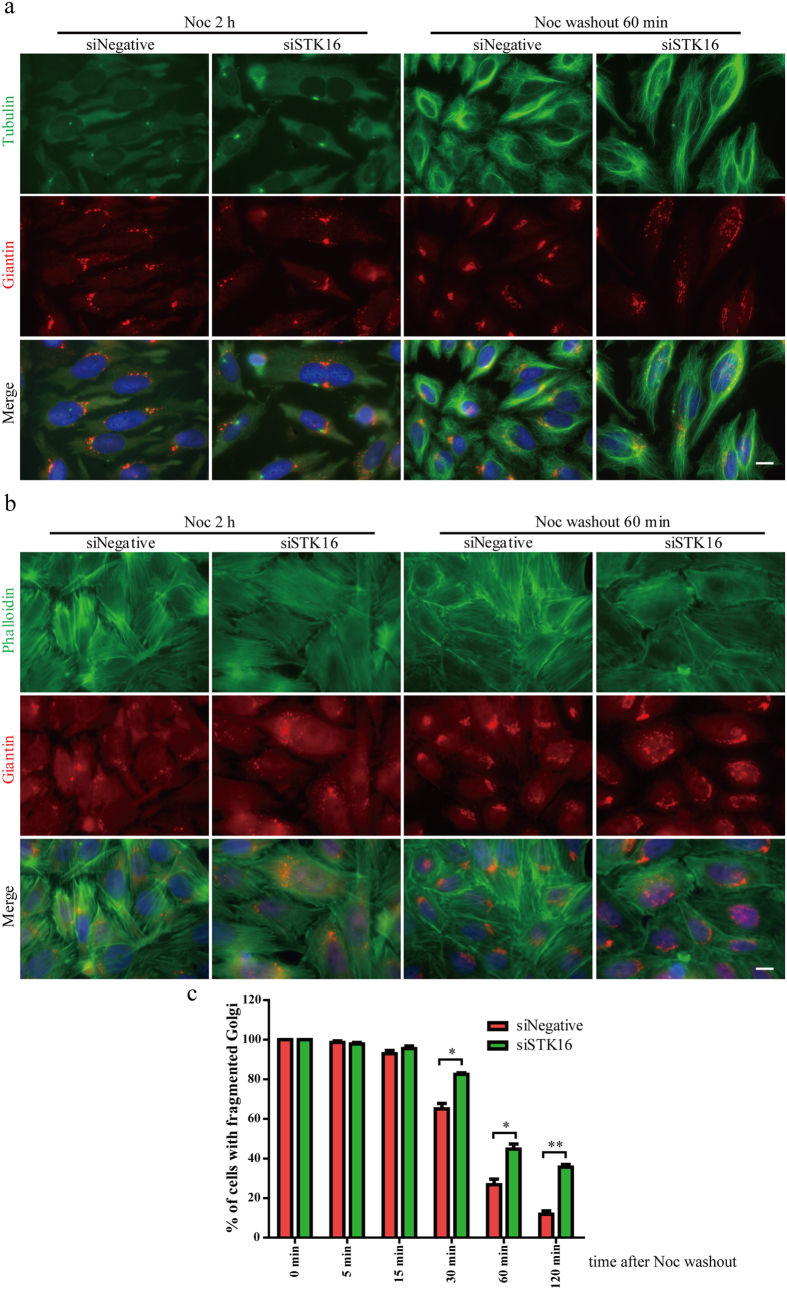
STK16 functions in Golgi reassembly. HeLa cells transfected with control or STK16 siRNAs for 72 hours were incubated with 10 μg/mL Nocodazole for 2 hours. After Nocodazole washout for 0, 5, 15, 30, 60, 120 minutes, cells were fixed and stained for tubulin (green, in (**a**)) or fluorescence-labeled phalloidin (green, in (**b**)) and Giantin (red). 0 and 60 minutes results are shown. (**c**) Quantification of the percentage of cells with fragmented Golgi in (**b**). Experiments were repeated two times and representative results are shown. The data were analyzed using a student’s *t*- test (*p < 0.05, **p < 0.01). Data show mean ± SEM, n = 2. Scale bar, 10 μm.

It has been shown that depolymerizing microtubules with Nocodazole results in Golgi ministacks dispersed in the cytoplasm^33^,^34^. We found that STK16 almost completely distributed from its original Golgi localization to scattered cytoplasmic dots after Nocodazole treatment (Supplementary Fig. S2a). In addition, the dispersed STK16 partially colocalizes with both Giantin and TGN46, which indicates that STK16 not only localizes to the Golgi, but also to TGN (trans-Golgi network) (Supplementary Fig. S2a). Similarly, when the Golgi was disassembled by Brefeldin A, STK16 was also dispersed (Supplementary Fig. S3). Furthermore, to determine whether STK16 participates in Golgi complex formation, we treated HeLa cells with 10 μg/mL Nocodazole for 2 hours and washed it away to allow the reformation of Golgi ribbon to mimic postmitotic Golgi complex assembly in the presence or absence of STK16 (Fig. 2 and Supplementary Fig. S4). We found that the reformation of the Golgi complex was significantly delayed in STK16 knockdown cells, whereas the reformation of microtubules was not affected (Fig. 2a). In control cells, after Nocodazole removal for 1 hour, the Golgi complex was mostly reformed, whereas the STK16 knockdown cells showed lots of diffused Golgi vesicles (Fig. 2a and b). In addition, the extent of microtubule regrowth and the Golgi structure reformation was time-dependent and it was much more complete at 60 minutes compared to 15 minutes after Nocodazole washout. Quantification results revealed that from 30 minutes of Nocodazole washout, at each time point, there were much more cells with fragmented Golgi in STK16-knockdown cells than in control cells (Fig. 2c). For example, at 60 minutes, the percentage of cells with fragmented Golgi in STK16 knockdown cells was about 45%, which was 15% more than control cells (p < 0.05). At 120 minutes after Nocodazole removal, about 90% of cells reformed the Golgi ribbon; while in STK16 siRNA-treated cells, there were still 35% of cells had diffused Golgi vesicles (p < 0.01) (Fig. 2c). This indicates that STK16 plays critical roles in Golgi reassembly.

### STK16 interacts with actin and regulates actin polymers in cells

**Figure 3:**
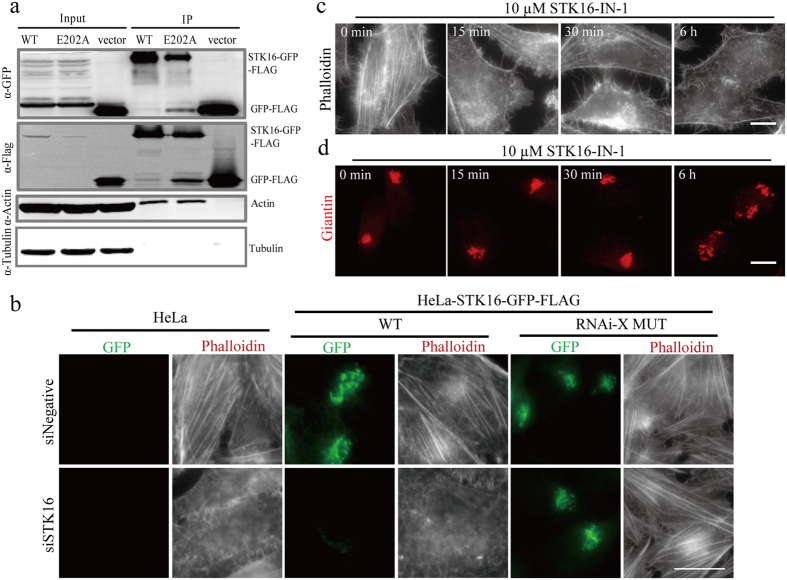
STK16 interacts with actin and regulates actin dynamics in cells in a kinase activity-dependent way. **(a**) Immunoprecipitation shows that STK16 interacts with actin and both wild-type and the kinase-dead mutant STK16 bind to actin equally. HeLa cells that stably express STK16-GFP-FLAG-WT, STK16-GFP-FLAG-E202A or GFP-FLAG were subjected to immunoprecipitation with anti-Flag antibodies. Anti-Actin, anti-Tubulin and anti-FLAG antibodies were used in Western blots. The input/IP loading ratio was 1/10. (**b**) STK16 depletion causes actin depolymerization *in vivo*. HeLa, HeLa-STK16-GFP-FLAG-WT (WT) and HeLa-STK16-GFP-FLAG-MUT (RNAi resistant mutant, RNAi-X MUT) cells were transfected with control or STK16 siRNAs for 72 hours before they were fixed and stained for GFP (green) and fluorescence-labeled phalloidin (red). (**c,d**) STK16-IN-1 disrupts actin stress fiber earlier than Golgi complex. HeLa cells were treated with 10 μM STK16-IN-1 for 0, 15, 30, 60 minutes (not shown here), or 6 hours before they were harvested for immunofluorescence using fluorescence-labeled phalloidin (**c**) or anti-Giantin antibody (**d**). Experiments were repeated at least three times and representative results are shown. Scale bar: 10 μm.

Since a dynamic actin network is necessary to maintain Golgi ribbon connection in mammalian cells^5^, we also examined actin in STK16 RNAi experiments in Fig. 2b. We found that the F-actin seemed to be affected by STK16 RNAi. In addition, since STK16-IN-1 can induce binucleated cells, which is generally caused by cytokinesis failure, we speculated actin might be involved in STK16 function in cells. We first conducted immunoprecipitation (IP) experiments using an anti-Flag antibody in HeLa cells stably expressing STK16-GFP-FLAG-WT (wild-type) or STK16-GFP-FLAG-E202A (kinase-dead mutant). Interestingly, both wild-type and the kinase-dead mutant STK16 could pull out actin, but not tubulin (Fig. 3a), which indicates that STK16 binds to actin in cells and the kinase activity of STK16 is not required for this binding. In addition, since it is well known that actin can be found in many IP experiments because it is abundant and sticky, we used the empty vector (GFP-FLAG) transfected cells as a negative control to exclude potential false positive results. Our data show that actin does not bind to vector control, which confirmed the specific binding between actin and STK16.

Next we tested whether actin cytoskeleton was affected by STK16 knockdown. RNAi experiments showed that STK16 knockdown decreased F-actin in HeLa and HeLa-STK16-GFP-FLAG-WT cells but not in the RNAi resistant mutant cell line HeLa-STK16-GFP-FLAG-MUT (Fig. 3b). In addition, STK16-IN-1 induced F-actin depolymerization in wild-type HeLa-STK16-GFP-FLAG but not the drug resistant mutant F100C (Supplementary Fig. S5). Moreover, we also tested STK16-IN-1 in mouse NIH-3T3 and human MCF-7 cells, and found that the actin cytoskeleton was also depolymerized in these cells (Supplementary Fig. S6). Taken together, these results showed that STK16 regulates actin polymers in cells and its kinase activity is important for actin regulation.

To test whether STK16 regulated Golgi complex integrity through affecting actin dynamics, we treated HeLa cells with STK16-IN-1 for different time points to see whether the actin polymers were affected earlier than the Golgi. Our results showed that 1 μM STK16-IN-1 started to depolymerize actin as early as in 30 minutes while the Golgi complex integrity showed no significant change even after 1 hour (Supplementary Fig. S7). More evidently, there were a lot of actin puncta when cells were treated with 10 μM STK16-IN-1 for only 15 minutes (Fig. 3c), while the Golgi was not affected until 4–6 hours (Fig. 3d). These results indicated that STK16 may regulate Golgi complex through affecting actin.

### *In vitro* assays show that STK16 directly regulates actin dynamics in a concentration- and kinase activity- dependent way

**Figure 4:**
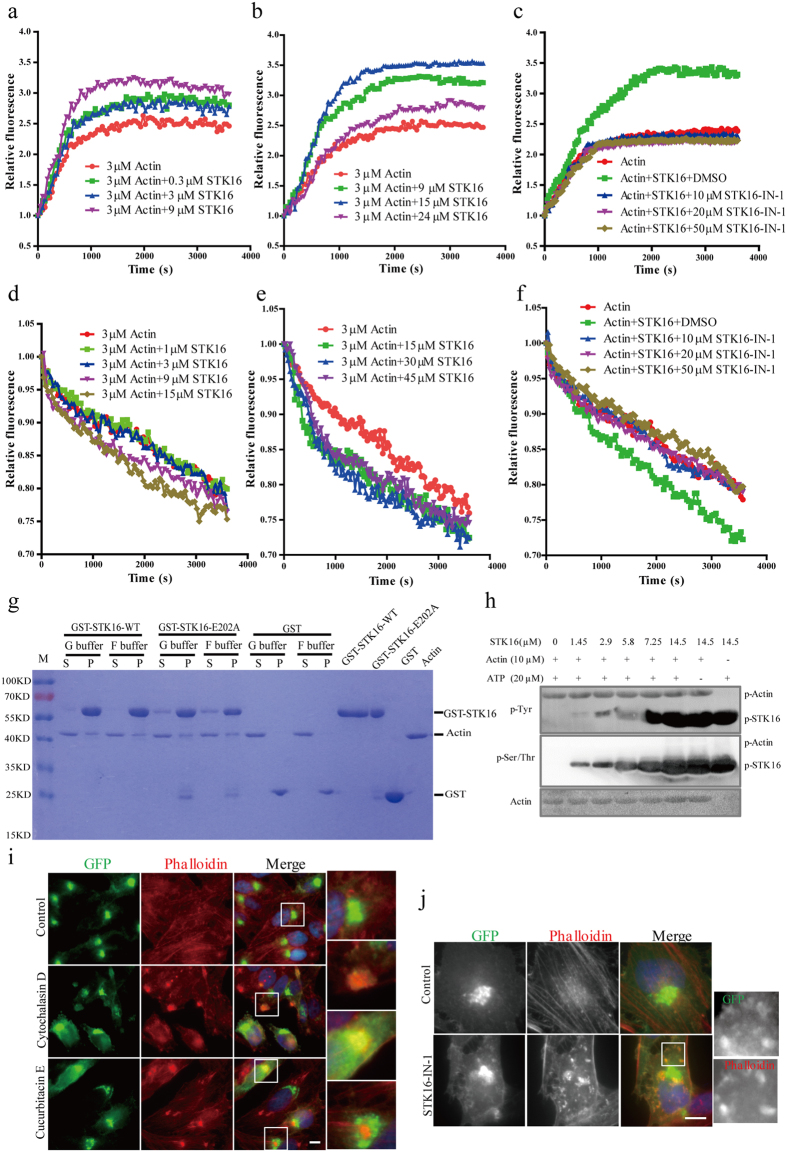
STK16 directly binds to actin and regulates actin dynamics *in vitro* in a concentration- and kinase activity -dependent way. (**a,b**) STK16 protein stimulates actin polymerization, and peaked at 5:1 ratio (STK16: actin). 3 μM G-actin mixed with 10% pyrene-actin was incubated with 0.3, 3 and 9 μM (**a**), or 9, 15, and 24 μM (**b**) STK16 protein. Polymerization was induced by 10× polymerization buffer. (**c**) The kinase activity of STK16 protein is critical for its stimulation effect on actin polymerization. 10 μM, 20 μM, and 50 μM STK16-IN-1 were incubated with 3 μM G-actin mixed with 10% pyrene-actin and 15 μM STK16 protein, respectively. (**d,e**) STK16 affects actin depolymerization at higher concentrations. F-actin polymerized from 3 μM G-actin mixed with 10% pyrene-actin was incubated with 1, 3, 9 and 15 μM (**d**), or 15, 30, and 45 μM (**e**) STK16 protein. Depolymerization was induced by adding 2 μM Latrunculin A. (**f**) The kinase activity of STK16 protein plays a role in actin depolymerization. 10 μM, 20 μM, and 50 μM STK16-IN-1 were incubated with F-actin polymerized from 3 μM G-actin mixed with 10% pyrene-actin and 30 μM STK16 protein, respectively. (**g**) GST pull-down experiments show that both WT and E202A STK16 bind to actin directly. GST, GST-STK16-WT or GST-STK16-E202A protein and actin were at 5:1 ratio in G-buffer or F-buffer, respectively. “S”, Supernatant; “P”, Pellet. (**h**) 0, 1.45, 2.9, 5.8, 7.25, 14.5 μM STK16 protein was incubated with 10 μM Actin. The phospho-Tyrosine, phospho-Serine/Threonine, and actin antibodies were used in western blots. (i) STK16 binds to actin puncta after actin depolymerization *in vivo*. HeLa-STK16-GFP-FLAG-WT cells treated with DMSO, 100 nM Cytochalasin D, and 10 nM Cucurbitacin E for 4 hours were stained for anti-GFP antibody (green) and fluorescence-labeled phalloidin (red). (**j**) STK16 partly distributes from its original localization to cytoplasm and binds with actin puncta after its kinase activity is inhibited by STK16-IN-1. HeLa-STK16-GFP-FLAG-WT cells were treated with DMSO and 50 μM STK16-IN-1 for 2 hours. Experiments were repeated 2–3 times and representative images are shown. Scale bar, 10 μm.

We next examined whether STK16 directly affects actin. STK16 protein was purified as previously described^29^, and subjected to pyrene-actin polymerization and depolymerization assays. As 1–5 μM actin monomer was used by recent studies on *in vitro* actin dynamics, we used 3 μM actin in this part of the experiments^35^,^36^,^37^,^38^. At 360/420 nm, the fluorescent signal of monomer pyrene actin (G-actin) was enhanced linearly after its polymerization into filaments during the first 15 minutes, while STK16 had no absorbance (Supplementary Fig. S8a). Moreover, there was no fluorescence increase before the polymerization buffer was added (Supplementary Fig. S8b). Our results show that STK16 started to increase actin polymerization from very low concentration, at one tenth of actin concentration (Fig. 4a) and reached its maximum capacity when it was used at 5-fold concentration of actin (Fig. 4b). It is interesting that at higher STK16 concentration (8-fold of actin), the actin polymerizing effect of STK16 was decreased (Fig. 4b). In addition, STK16-IN-1 effectively inhibited the polymerizing effect of STK16 (Fig. 4c), which not only proves the specific effect of STK16 protein itself on actin polymerization, but also indicates that the kinase activity of STK16 is required for its function to polymerize actin.

We next examined the effect of purified STK16 on actin depolymerization. 2 μM Latrunculin A was incubated with F-actin to induce its depolymerization before different concentrations of STK16 protein were added (Fig. 4d and e). It is interesting that at low concentrations of STK16, one third or equal amount of actin, did not affect actin depolymeriziation (Fig. 4d). However, at higher concentrations, from 3:1 to 15:1 molar ratio to actin, STK16 effectively increased actin depolymerization (Fig. 4e). In addition, the actin depolymerization activity could also be inhibited by STK16-IN-1 (Fig. 4f).

Therefore, our *in vitro* experiments show that STK16 protein can directly regulate actin dynamics in a concentration and kinase activity dependent manner. Lower concentrations of STK16 tend to increase actin polymerization while higher concentrations of STK16 promote actin depolymerzation. Since the small molecule inhibitor STK16-IN-1 inhibited its activity in both actin polymerization and depolymerzation, the kinase activity of STK16 seems to be required for its regulation activity on actin dynamics.

### STK16 directly binds to actin and the E202A mutation does not affect its actin-binding ability

To test the possibility of the direct binding of STK16 to actin, we expressed and purified GST-STK16 protein and conducted *in vitro* pull-down assays with G-actin and F-actin, respectively. As shown in Fig. 4g, actin could weakly but directly bind to GST-STK16 (Fig. 4g). Again, to exclude potential false positive results, we used the GST protein as the negative control and found that actin did not bind to the GST negative control (Fig. 4g), confirming the specificity between STK16 and actin *in vitro*. In addition, GST-STK16-E202A could also bind to actin, which is consistent with our previous IP results using cell lysate. Since it was reported that phosphorylation of actin is critical for its dynamics^39^,^40^,^41^,^42^, we wanted to find out whether actin could be phosphorylated by STK16. Our results showed that STK16 had no effect on tyrosine phosphorylation in actin (Fig. 4h). In addition, STK16 did not phosphorylate the serine/threonine residues in actin either (Fig. 4h).

Then we further tested the interaction of STK16 and actin in cells. Cytochalasin D (Cyto D) is known to cap actin filament and therefore inhibits actin filament elongation^43^. Cucurbitacin E (CurE) specifically binds to and depolymerizes F-actin and induces actin aggregations^38^. In Cyto D and CurE treated cells, we found STK16 partly diffused from its original localization to cytoplasm, some of which colocalized with actin aggregates (Fig. 4i). Moreover, when we treated HeLa cells stably expressing STK16-GFP-FLAG-WT with the inhibitor STK16-IN-1 for 2 hours, there were also some STK16 disassociation from its original localization and formation of some cytoplasmic aggregation that colocalized with actin (Fig. 4j). These results confirmed the association between STK16 protein and actin in cells.

### STK16 knockdown or kinase inhibition does not affect VSVG trafficking from endoplasmic reticulum (ER) to plasma membrane (PM)

**Figure 5:**
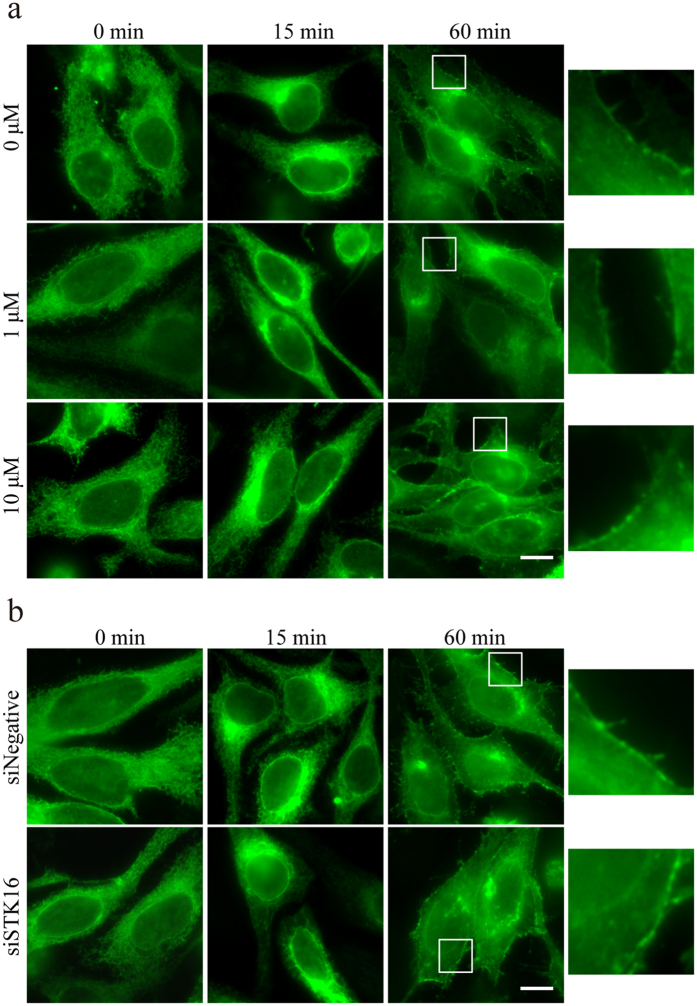
STK16 kinase inhibition or knockdown does not affect VSVG trafficking from ER to PM. (**a**) The kinase activity of STK16 is not required for VSVG-GFP trafficking. HeLa cells were transfected with VSVG-GFP plasmids for 24 hours, then treated with DMSO, 1 μM or 10 μM STK16-IN-1 at 40 °C overnight. After shifted to 33 °C for 15 or 60 minutes, cells were fixed and stained by the GFP antibody to show the VSVG-GFP protein arrival at Golgi or PM, respectively. The right panel shows an enlarged view of boxed region in the left panel. (**b**) STK16 knockdown does not affect VSVG-GFP trafficking. HeLa cells were transfected with control or STK16 siRNAs for 48 hours before they were transfected with VSVG-GFP plasmids for 24 hours. Cells were incubated at 40 °C overnight, and shifted to 33 °C for 15 or 60 minutes, and then harvested for immunofluorescence using the GFP antibody. The right panel shows an enlarged view of boxed region in the left panel. Experiments were repeated two times and representative images are shown. Scale bar, 10 μm.

To investigate the function of STK16 in the Golgi related membrane trafficking, we examined the transport of GFP-tagged version of the temperature sensitive viral protein VSVG^44^. VSVG-GFP transfected HeLa cells were treated with DMSO or 10 μM STK16-IN-1, incubated at 40 °C overnight and followed by 33 °C incubation. The VSVG-GFP protein misfolds and is retained in the ER at 40 °C, but upon temperature shift to 33 °C, it moves to the Golgi complex before being transported to the PM. In both DMSO and STK16-IN-1 treated cells, the VSVG-GFP protein was retained in the ER at 0 min (Fig. 5a). After the temperature shifts to 33 °C for 15 minutes, the VSVG-GFP protein was transported to the Golgi in both DMSO and STK16-IN-1 treated cells. This indicated that the ER to Golgi transport of VSVG-GFP was not affected by STK16 kinase inhibition (Fig. 5a). After the temperature shifted to 33 °C for 60 minutes, the VSVG-GFP protein arrived at the PM, and there was no difference between DMSO and STK16-IN-1 treated cells (Fig. 5a), which suggested that the Golgi to PM transport of VSVG-GFP was not affected by STK16 kinase inhibition either. Then we analyzed whether STK16 protein itself is required for VSVG transport by STK16 RNAi. Our results showed that the delivery of VSVG from ER to Golgi and then to PM was not affected by STK16 knockdown either (Fig. 5b). Therefore, the kinase activity of STK16 and the STK16 protein itself are not required for the ER-Golgi-PM transport of VSVG.

### STK16 knockdown or kinase inhibition arrests cells in G2 phase, prometaphase and cytokinesis

**Figure 6:**
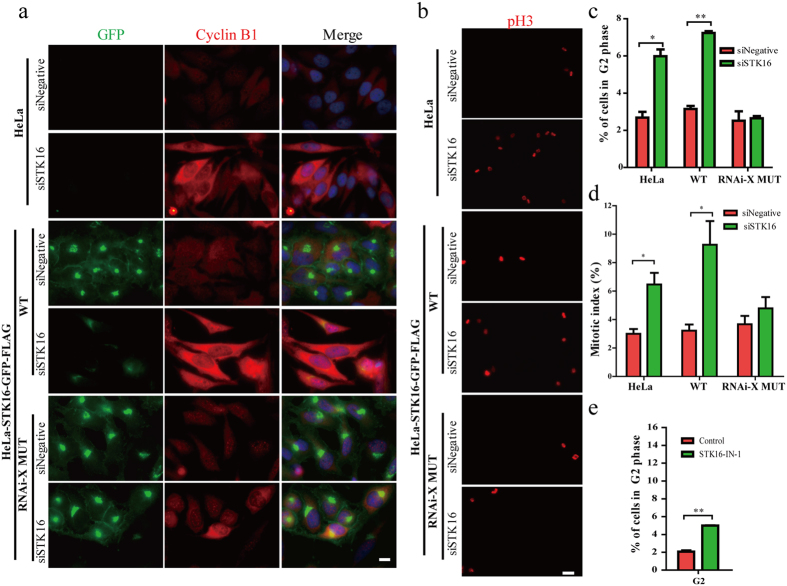
STK16 depletion or kinase activity inhibition increases G2/M cell percentage. (**a,b**) STK16 depletion increased cells that are positively stained with cyclin B1 and phospho-H3. HeLa cells, as well as HeLa cells stably expressing STK16-GFP-FLAG-WT or MUT (RNAi resistant mutant) were transfected with control or STK16 siRNAs for 72 hours, then they were fixed and stained for anti-GFP (green) and anti-Cyclin B1 (red) antibodies in (a) or with phospho-H3 in (**b**). (**c**) Quantification of percentage of G2 phase cells in (**a**). (**d**) Quantification of mitotic index after STK16 knockdown in HeLa, HeLa-STK16-GFP-FLAG-WT, and HeLa-STK16-GFP-FLAG-MUT cells. (**e**) Inhibition of STK16 kinase activity increases cells in G2 phase. HeLa cells were treated with 10 μM STK16-IN-1 for 6 hours, then fixed for immunofluorescence using anti-Cyclin B1 antibody and DAPI. Quantification of percentage of G2 stage cells was shown. The data were analyzed using a student’s *t*- test (*p < 0.05, **p < 0.01). Data show mean ± SEM, n = 3. Scale bar, 10 μm.

During the mammalian cell cycle, the well regulated multi-step Golgi disassembly and reassembly are critical events for both mitotic entry as well as mitotic progression^18^,^20^,^45^. We previously found that STK16 knockdown or kinase activity inhibition both led to increased G2/M phase population in flow cytometry analysis^29^, which we now hypothesis to be at least partially due to the Golgi disturbance. However, since flow cytometry analysis does not differentiate the G2 phase from mitosis, here we used immunofluorescence experiments to examine whether the mitotic index (% of cells in mitosis, from prophase to telophase) is affected by STK16 perturbation. It is interesting that siSTK16 treatment increased the number of cells with a large nucleus, which were positively stained with cyclin B1 (Fig. 6a). This indicates that they are in the G2 phase. It is obvious that the percentage of G2 phase cells was increased in both HeLa and HeLa-STK16-GFP-FLAG-WT cell lines but not in the RNAi resistant mutant cell line by STK16 RNAi knockdown (Fig. 6a). Furthermore, the percentage of the cells positively stained with phospho-Histone H3 was also increased by STK16 RNAi knockdown in HeLa and HeLa-STK16-GFP-FLAG-WT cell lines but not in the RNAi resistant mutant cell line (Fig. 6b). We further quantified these experiments and found that the percentage of G2 phase cells in HeLa and HeLa-STK16-GFP-FLAG-WT, but not in the RNAi resistant mutant, was increased by almost 3-fold by STK16 knockdown (Fig. 6c). The mitotic index was also significantly increased by STK16 RNAi (Fig. 6d). In addition, STK16-IN-1 also increased Cyclin B1 positive cells, which could be rescued by the drug-resistant mutant F100C (Supplementary Fig. S9). Quantification showed that STK16-IN-1 increased both cells in G2 phase (Fig. 6e) and mitosis (Supplementary Fig. S10), which is similar to the STK16 RNAi results.

**Figure 7:**
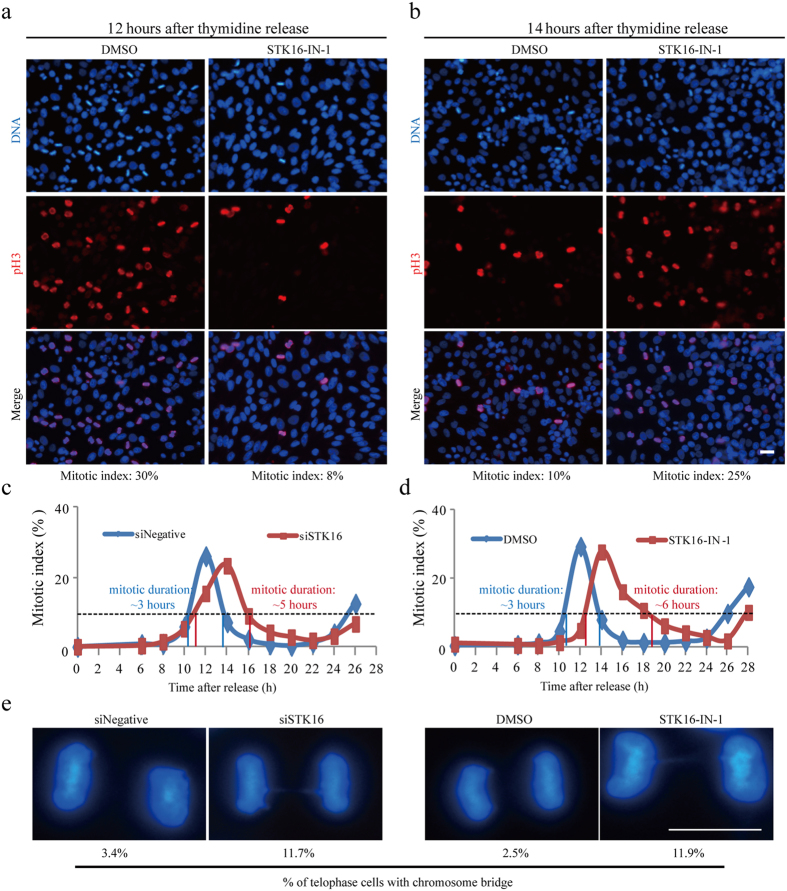
STK16 depletion or kinase activity inhibition delays mitotic entry and causes prolonged mitosis. HeLa cells were plated and treated with siRNA or STK16-IN-1 and then subjected to double thymidine synchronization. (**a,b**) Cells were released from thymidine for 12 or 14 hours before they were harvested for immunofluorescence. (**c,d**) The percentage of cells in mitosis (mitotic index) was determined by staining DNA with DAPI and an anti-phospho-Histone H3 antibody at 0, 6, 8, 10, 12, 14, 16, 18, 20, 22, 24, 26 and 28 hours after thymidine release for control and STK16 siRNAs transfected cells, or for DMSO and STK16-IN-1 treated cells. For each time point, >1000 cells were counted. Data show mean value, n = 2. Experiments were repeated two times and representative images are shown. (**e**) STK16 knockdown or inhibition increases cells with chromosome bridges. Representative images of DNA in siNegative, siSTK16, or in DMSO or STK16-IN-1 treated cells. Scale bar, 10 μm.

To get more details of these STK16 knockdown and kinase inhibition-induced cell cycle defects, we synchronized HeLa cells with double thymidine and used RNAi or STK16 inhibitor to examine the cell cycle progression (Fig. 7). We found that 12 hours after the second thymidine release, there were much less mitotic cells in STK16-IN-1-treated HeLa cells than the control group (Fig. 7a). While at 14 hours after the second thymidine release, there were much more mitotic cells in STK16-IN-1-treated HeLa cells than in the control group (Fig. 7b). We then did some detailed time points analysis to examine the whole cell cycle progression in both STK16 knockdown (Fig. 7c) and kinase inhibitor (Fig. 7d) treated HeLa cells. Quantification results showed that the mitotic entry was significantly delayed by both STK16 knockdown and kinase inhibition. In addition, the whole mitotic duration was also prolonged by both STK16 knockdown and kinase inhibition. If we use 10% of mitotic index as a threshold, the mitosis in control cells was around 3 hours while the STK16 knockdown and kinase inhibition prolonged it to 5 and 6 hours, respectively (Fig. 7c and d). Interestingly, we also found that there were increased telophase cells with chromosome bridges in STK16 perturbed cells (Fig. 7e). STK16 knockdown or kinase inhibition increased the percentage of telophase cells with chromosome bridges by 3–4 fold (Fig. 7e).

**Figure 8:**
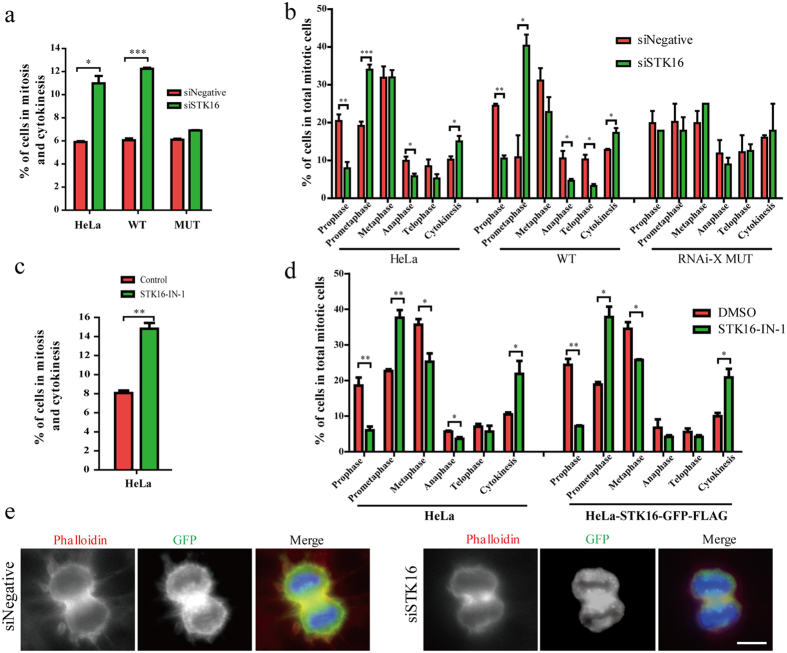
STK16 affects mitotic progression. (**a,b**) Quantification of cells in mitosis and cytokinesis, cells in each cell cycle stage during mitosis and cytokinesis in HeLa, HeLa-STK16-GFP-FLAG-WT (WT), and HeLa-STK16-GFP-FLAG-MUT (RNAi resistant mutant, RNAi-X MUT) cells transfected with siSTK16. (**c,d**) Quantification of cells in mitosis and cytokinesis, cells in each cell cycle stage during mitosis and cytokinesis in HeLa and HeLa-STK16-GFP-FLAG-WT cells treated with 5 μM STK16-IN-1 for 72 hours. (**e**) Actin spikes and membrane blebbing in cytokinesis cells are reduced after STK16 knockdown. HeLa cells stably expressing STK16-GFP-FLAG-WT were transfected with control or siSTK16 for 3 days before they were fixed and stained by phalloidin and anti-GFP. Experiments were repeated three times and representative images are shown. The data were analyzed using a student’s *t*- test (*p < 0.05, **p < 0.01, ***p < 0.001). Data show mean ± SEM, n = 3. Scale bar, 10 μm.

**Figure 9:**
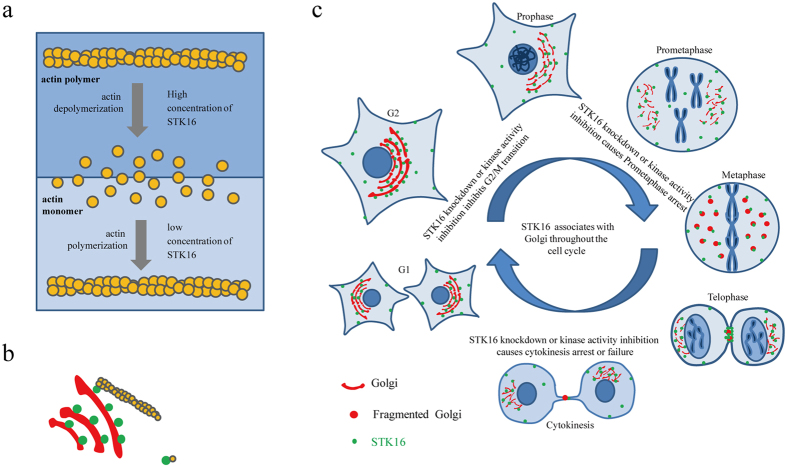
The model of STK16 functions in actin, the Golgi and cell cycle regulation. (**a**) STK16 kinase is a novel actin binding protein that regulates actin polymerization and depolymerization in a concentration–dependent manner. (**b**) STK16 localizes to the Golgi and bridges the Golgi with actin. (**c**) STK16 localizes to the Golgi and plays important roles in G2/M transition, mitotic progression as well as cytokinesis.

The prolonged mitosis we observed indicates that the mitotic progression was affected by STK16 knockdown and kinase inhibition. We quantified the mitotic index in HeLa, HeLa-STK16-GFP-FLAG wild-type, and STK16 RNAi resistant mutant cells, with or without siSTK16 treatment. We found that the cells in mitosis and cytokinesis are significantly increased by STK16 knockdown (Fig. 8a). Then we further looked into more details by quantifying cells in each phase, from prophase to cytokinesis, and found that STK16 knockdown increased the cell populations in both prometaphase and cytokinesis (Fig. 8b). In addition, STK16-IN-1 also had similar effects as STK16 knockdown (Fig. 8c and d). Since reorganization of the actin cytoskeleton for cytokinesis is crucial^46^,^47^,^48^, we analyzed the actin cytoskeleton in late mitosis. We found that STK16 knockdown cells had strikingly reduced actin spikes and membrane blebbing compared to control (Fig. 8e), which are critical for furrow ingression and abscission during cytokinesis. Therefore STK16 regulates actin dynamics, Golgi assembly, and plays critical roles in mitotic entry, mitotic progression, and cytokinesis (Fig. 9).

## Discussion

The rapid assembly and disassembly of actin monomers (G-actin) into filaments (F-actin) is critical to various cell processes^49^. In this study, we identified STK16 as a new actin interacting protein by both *in vitro* pull down and immunoprecipitation experiments. Although there were some other serine/threonine kinases also located in the Golgi complex and play important roles in Golgi regulation, STK16 is the first one that can bind to actin and regulates actin dynamics directly. The others, such as Cdc42^50^,^51^ and PAK4^52^, participate in actin dynamics regulation through cell signaling pathways or interacting with actin binding proteins. PKD1/PKD2 distributed in the Golgi apparatus do not influence the actin polymerization or affect F-actin^53^. Thus, our study reveals a serine/threonine kinase residing in Golgi can regulate actin dynamics through binding to actin directly.

Although our data show that STK16 is not critical for ER to Golgi or Golgi to PM transport of VSVG, the Golgi structure and cell cycle were obviously affected by STK16 perturbation. The cell cycle effects of STK16 knockdown and kinase inhibition are likely due to the actin dynamics regulation by STK16. It has been shown that depolymerization of actin filaments by toxins can delay mitotic progression in primary cells and fission yeast^54^,^55^. F-actin was found to surround the mitotic spindle, extend between the spindle and the cortex, and play antagonistic roles in spindle length maintenance^56^. Moreover, disruption of the cortical actin architecture during mitosis can severely affect spindle orientation, causing centrosome separation and spindle assembly abnormalities^57^,^58^. Since STK16 can directly bind to actin and regulates its dynamics, we hypothesize that the prolonged mitosis and prometaphase arrest in cells with STK16 knockdown and kinase inhibition are likely due to the altered actin dynamics during mitotic progression.

STK16 directly regulates actin polymerization and depolymerization in a concentration-dependent manner, which is similar to profilin and cofilin. Similar to STK16, profilin promotes actin polymerization at lower concentration but promotes actin depolymerization at higher concentration. Specifically, profilin binds to the barbed end of an actin monomer and dissociates rapidly to free the end for further elongation. However, high concentrations of free profilin can slow down elongation and even promote dissociation of the terminal subunit^59^,^60^. In contrast, cofilin severs actin filaments at low concentration, but nucleates actin assembly at high concentration^61^. In cells, many proteins control both the assembly and disassembly of actin filaments and they compete with each other for binding actin monomers or F-actin to regulate actin dynamics simultaneously. Depending upon the local environment, the newly formed ends of the severed filaments may nucleate filament growth or may increase subunit dissociation and filament depolymerization^62^,^63^. We speculate that under physiological conditions, the cellular actin monomer concentration is much higher than STK16, so STK16 mostly plays a critical part in actin polymerization. When the local concentration of STK16 is high, such as in the Golgi, STK16 starts to promote F-actin depolymerization. Further investigation is definitely needed to unravel the detailed exact mechanism of STK16 regulation on actin dynamics and the Golgi complex.

It is interesting that the kinase activity of STK16 is apparently critical for its activity in actin dynamics regulation, but not for its binding to actin. However, this is not entirely unexpected because the actin dynamics regulation effect is not always correlated with the binding affinity. For example, 10 nM S. pombe cofilin bound to only 0.12% of actin subunits and severing was maximal while 1 mMS. pombe cofilin bound to 49% of actin subunits but no severing was observed^61^. The binding affinity of STK16 to actin is not very strong, as shown in the immunoprecipitation and pull down assays, but the actin dynamics regulation by STK16 is unambiguous. In addition, our results indicate that the actin binding ability of STK16 is not the determining factor for its Golgi localization; the E202 mutant of STK16 can bind to actin but does not localize to the Golgi. The detailed mechanisms of local regulation of actin dynamics at the Golgi, as well as the regulation mechanisms, including its autophosphorylation, upstream factors, and downstream substrates, all need further investigations.

In conclusion, here in this study, we have identified STK16 kinase as a novel actin binding protein that resides in the Golgi and regulates actin polymerization and depolymerization in a concentration and kinase activity–dependent manner. STK16 localizes to the Golgi throughout the cell cycle and regulates Golgi assembly by interacting with actin, through which it plays important roles in G2/M transition, mitotic progression, as well as cytokinesis.

## Materials and Methods

### Cell culture

HeLa, MCF-7, and NIH-3T3 cells were grown in DMEM without L-glutamine (Corning Life Sciences), supplemented with 10% (v/v) fetal bovine serum (Clark), 1% (v/v) penicillin/streptomycin (Hyclone), 1% glutamax (Gibco), 5% CO_2_, at 37 °C. HeLa-STK16-GFP-FLAG wild type (WT), HeLa-STK16-GFP-FLAG RNAi resistant mutant (RNAi-X MUT), HeLa-STK16-GFP-FLAG E202A (E202A), HeLa-STK16-GFP-FLAG F100C (F100C) were maintained similar to HeLa cells, with addition of 1 μg/ml puromycin (Invitrogen) in DMEM.

### Wild-type, RNAi-resistant mutant, F100C, and E202A STK16-GFP-FLAG stable cell lines

The cDNA encoding human STK16 was cloned into a pMSCV-puro vector with GFP and 3 × FLAG tag fused at the C-terminal to form STK16-GFP-FLAG wild type (WT) plasmid. RNAi-X MUT plasmid was constructed by silent mutagenesis at the 894^th^–914^rd^ region GGCCAACATACTACCCAAATC → GGTCAGCACACGACTCAGATA with 37% mismatch. STK16-GFP-FLAG F100C (F100C) and E202A plasmids were point mutated by replacing the 299^th^ nucleotide T by G, and the 605^th^ nucleotide A by C, individually. Retroviruses were packaged by transfecting the four plasmids with two helper plasmids into 293 cell lines using Fugene 6 (Promega). Then the supernatants were harvested after 48 hours of incubation and infecting the HeLa or MCF-7 cells. Stable cell lines were screened by puromycin and maintained in a culture medium containing a final concentration of 1 μg/ml puromycin.

### RT-PCR

Total RNA was extracted following a modified guanidinium thiocyanate method using Trizol (Invitrogen) after cells were washed with phosphate buffered saline (PBS). cDNA was synthesized using PrimeScript^®^ RT reagent Kit (Takara). STK16, GAPDH and ACTIN genes were amplified from prepared cDNAs using fast pfu DNA polymerase with the following primers. DNA levels were normalized to the matching densitometric value of the internal control GAPDH by ImageJ software.

STK16F: CTATGTGGACCTAGTGGAAGGGTTACAT

STK16R: CTTTGTCCTTCAGCCTTTCTATCTCATT

GAPDHF: GTCACCAGGGCTGCTTTTAACTCT

GAPDHR: GGGTCTCTCTCTTCCTCTTGTGCT

ACTINF: AGATCATGTTTGAGACCTTCAACACC

ACTINR: GCAATGATCTTGATCTTCATTGTGC

### Immunofluorescence

HeLa, HeLa-STK16-GFP-FLAG WT, RNAi-X MUT, E202A, and F100C cells were grown on coverslips in 24-well plates and treated with the siRNAs or drugs for indicated time points. Cells were fixed by −20 °C methanol for 5 minutes or 4% formaldehyde at room temperature for 20 minutes. Then coverslips were washed with TBS-Tx (TBS supplemented with 0.1% Triton X-100) and blocked by AbDil-Tx (TBS-Tx with 2% BSA and 0.05% sodium azide) at room temperature for at least 30 minutes. Coverslips were stained with primary antibodies then probed with the secondary fluorescently conjugated antibodies. To visualize F-actin fibers, cells were directly stained with Alexa Fluor^®^ 488 Phalloidin or Alexa Fluor^®^ 594 Phalloidin (Invitrogen). After being rinsed thoroughly by TBS-Tx, coverslips were mounted in anti-fade prolong Gold with DAPI. The primary antibodies used in immunofluorescence experiments were as follows: GFP (Abcam, 1:500), Flag (Sigma, 1:1000), Giantin (Abcam, 1:500), phospho-Histone H3 (Cell signaling, 1:1500), and Cyclin B1 (Santa Cruz, 1:100). Images were taken using a Leica MI4000B fluorescent microscope. All experiments were repeated at least three times and representative micrographs are shown in the Figures.

### Western blotting

Cells plated on 12-well plates were lysed on ice with the M-PER lysis buffer supplemented with a protease and phosphatase inhibitor cocktail (Roche) at 4 °C. The whole cell lysate was thermally denatured at 95 °C for 8 minutes with 2 × SDS loading buffer (20 mM Tris-HCl pH 8.0, 100 mM DTT, 2% SDS, 20% Glycerol, and 0.016% Bromophenol Blue) thoroughly, and electrophoresed on 12% SDS-PAGE gels, which were then transferred onto the PVDF membranes by Thermo Scientific Owl VEP-2. The membranes were blocked, then incubated with corresponding primary antibodies, including GFP (1:1000), Flag (1:1000), p-Tyr (BD, 1:1000), p-Thr/Ser (Abcam, 1:1000), Tubulin (1:2000) and Actin (1:2000). HRP-linked secondary antibodies (1:5000) were then incubated and visualization was performed using enhanced chemiluminescence Kits (Millipore or Thermo Scientific) and the blots were analyzed using Tanon Fine-do X6 (Tanon). Protein levels were normalized to the matching densitometric value of the internal control α-tubulin by ImageJ software.

### RNAi

HeLa, HeLa-STK16-GFP-FLAG WT, and RNAi-resistant mutant cells were plated in 12-well or 24-well plates. SiRNA for STK16 or negative control were transiently transfected using Hiperfect following the manufacturer’s protocol at 40 nM. The sequences of siSTK16 and siNegative oligos were GGCCAACAUACUACCCAAAU and UUCUCCGAACGUGUCACGUTT. After 72 hours of incubation, cells were either lysed for Western Blotting or fixed to perform immunofluorescence experiments.

### Co-immunoprecipitation

HeLa-GFP-FLAG, HeLa-STK16-GFP-FLAG WT, and E202A cells were cultured in 150 mm dishes to 95–100% confluence and lysed as above. The whole cell lysate was centrifuged at 4 °C, 13,000 g for 10 minutes. The supernatants were mixed with Flag antibody pre-conjugated Dynabeads protein G at 4 °C for 2 hours on a rotator. After washing with ice-cold-PBST (PBS with 0.05% Tween-20) three times (every 5 minutes per time) at 4 °C on a rotator, the Dynabeads were collected using the magnet. And the Dynabeads-Flag-protein mixture was supplemented with lysis buffer and 5 × SDS loading buffer followed by boiling at 95 °C for 8 minutes. The cell lysate after centrifuging was used as the input control. Western blotting was carried out to analyze the co-immunoprecipitation results.

### STK16 wild type and E202A protein expression

STK16 (residues 1–305) (GenBank accession number gi4505837) wild type protein for *in vitro* phosphorylation and pyrene-actin assay was expressed as previously described^29^. For GST-STK16 WT protein, human STK16 cDNA was subcloned into a pGEX-T vector, expressed in *E. coli* (BL21 [DE3]) cells and purified by glutathione affinity resin. GST-STK16 E202A protein was point mutated by replacing the 202^nd^ residue E by A and cloned into the same vector, followed by purification with slight modification.

### Pyrene-actin assay

Actin polymerization and depolymerization experiments were performed with 3.0 μM rabbit skeletal muscle actin mixed with 10% pyrene-actin. The actin mixture was diluted in G-buffer (0.2 mM CaCl_2_, 0.2 mM ATP, 5 mM Tris-HCl (pH 8.0), 1 mM DTT) and incubated for 1 hour on ice to depolymerize actin oligomers and subsequently centrifuged at 14,000 rpm at 4 °C for 15 minutes. For polymerization assays, supernatants were added to a 96-well black plate already containing different concentrations of STK16 protein. Then, the 96-well plate was placed into the fluorescent spectrophotometer and the samples were read for 3 minutes to establish a baseline fluorescent measurement. Polymerization was induced by the addition of 10х polymerization buffer (20 mM MgCl_2_, 500 mM KCl, 10 mM ATP), followed by incubation at room temperature for 1 hour. For depolymerization assays, supernatants were prepared as described above. Supernatants were then polymerized with 10х polymerization buffer for 1 hour, and subsequently centrifuged at 100,000 rpm for 30 min to isolate filamentous actin in the pellet from remaining monomeric actin in the supernatant. Filamentous actin was suspended again in an F-buffer (G-buffer + 10х polymerization buffer (1/10^th^ the volume of G-buffer)) and incubated with different concentrations of STK16 protein. Depolymerization was induced by the addition of 2 μM Latrunculin A (Sigma). The kinetics of polymerization and depolymerization were monitored at room temperature using Molecular Device SPECTRAMAX I3X with excitation and emission wavelengths of 360 nm and 420 nm, respectively.

### *In vitro* phosphorylation assay

Kinase assays were performed for 30 minutes at 37 °C in a final volume of 30 μl consisting of the kinase buffer (50 mM Tris, pH 7.4, 20 mM MgCl_2_, 2.5 mM DTT, 20 μM ATP) and 10 μM rabbit skeletal muscle actin as substrate. Reactions were stopped by the addition of 5х the sample buffer and boiled for 8 minutes. Samples were subsequently analyzed by SDS-PAGE and western blotting.

### *In vitro* pull down assay

GST, GST-STK16 WT, and GST-STK16 E202 proteins diluted in a binding buffer (20 mM Tris, 300 mM NaCl, pH 8.0) were first conjugated with glutathione resins respectively at 4 °C overnight on a rotator. Then, half of the resins were incubated with rabbit skeletal muscle actin in G-buffer, while the others incubated with rabbit skeletal muscle actin in F-buffer, at 4 °C for 2 hours on a rotator. Supernatants were preserved and the pellets were washed with G/F buffer supplemented with 0.1% Triton X-100 and suspended again with the G/F buffer. 5х the sample buffer was added to supernatants or pellets and boiled for 8 minutes. Samples were subsequently analyzed by SDS-PAGE.

### Cell synchronization assay

HeLa cells were seeded at 30% confluence in 24-wells plates with coverslips and synchronized by a double thymidine block. DMEM with 2.5 mM thymidine was added to cells for 16 hours. After being washed three times with PBS to remove thymidine, cells were incubated with fresh DMEM medium for 8 hours. Then DMEM medium containing 2.5 mM thymidine was added for another 16 hours for the 2^nd^ thymidine block to reserve cells at G1/S transition. “0” indicated the beginning of double thymidine release.

When double thymidine block was performed along with siRNA transfection, the first RNAi transfection was performed in suspension cells prior to seeding cells onto culture plates. The second RNAi transfection was performed after washing off the first batch of thymidine, along with fresh DMEM medium. The two batches of thymidine were added directly to the medium to avoid washing the siRNAs off.

When synchronization assays were done along with the STK16 inhibitor, DMSO and STK16-IN-1 were added 4 hours after the second thymidine release. Synchronized cells were collected at indicated times after being released for fixing and staining for antibodies.

## Additional Information

**How to cite this article:** Liu, J. *et al*. STK16 regulates actin dynamics to control Golgi organization and cell cycle. *Sci. Rep.* **7**, 44607; doi: 10.1038/srep44607 (2017).

**Publisher's note:** Springer Nature remains neutral with regard to jurisdictional claims in published maps and institutional affiliations.

## Supplementary Material

Supplementary Information
