# Supplementary material for: STK16 regulates actin dynamics to control Golgi organization and cell cycle
Source: Sci Rep. 2017 Mar 15;7:44607. doi: 10.1038/srep44607 (PMC5353726; doi:10.1038/srep44607)
Supplement: Supplementary Information [file srep44607-s1.pdf]

Supplemental Materials

STK16 regulates actin dynamics to control Golgi organization and cell cycle

Juanjuan Liu[1,2,3], Xingxing Yang[1,3], Binhua Li[1], Junjun Wang[1,2], Wenchao Wang[1], Jing Liu[1], Qingsong Liu[1,2], Xin Zhang[1]

[1] High Magnetic Field Laboratory, Chinese Academy of Sciences, Hefei, Anhui, 230031, P. R. China;
[2] University of Science and Technology of China, Hefei, Anhui, 230036, P. R. China.
[3] Co-first author.
Correspondence and requests for materials should be addressed to X. Z. (xinzhang@hmfl.ac.cn)

Figure S1

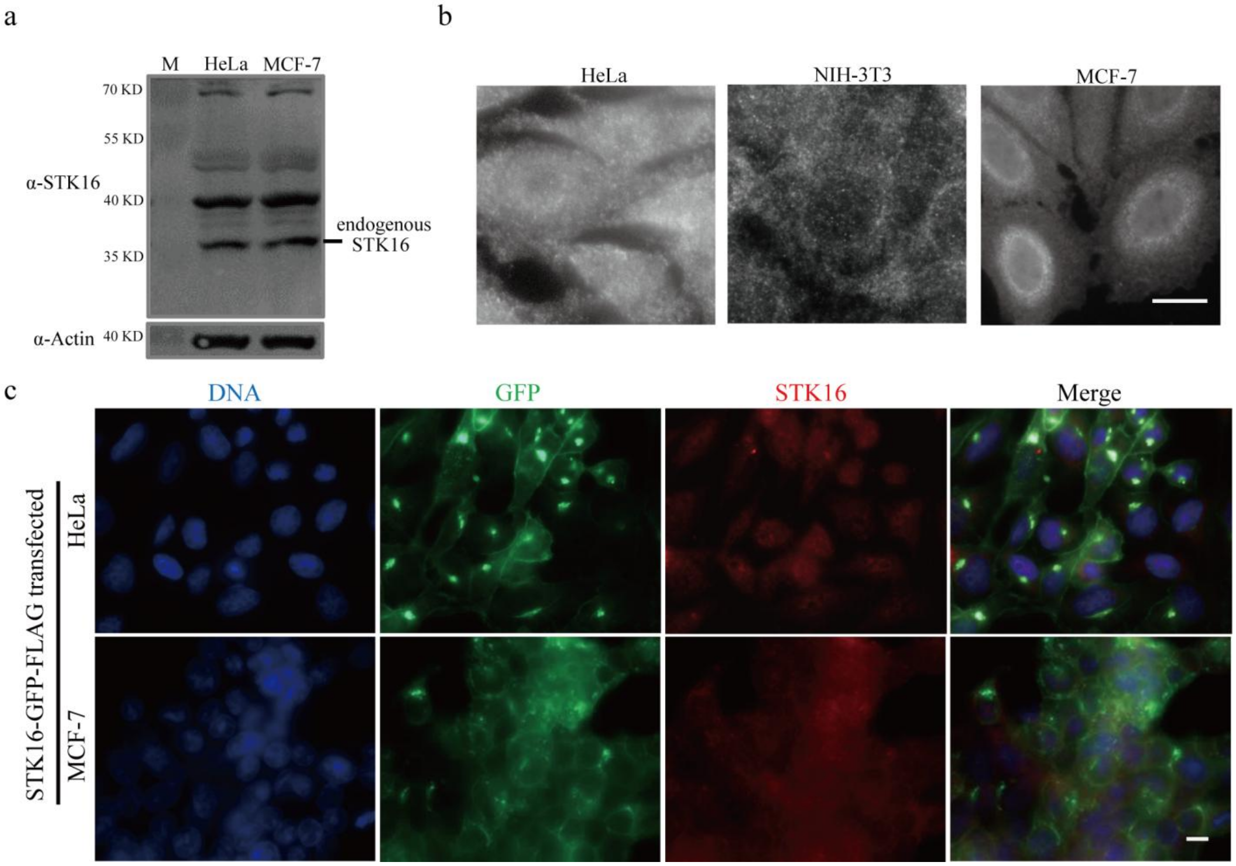

Figure S1. The anti-STK16 antibody we bought from Sigma does not work well in western blots and immunofluorescence. (a) HeLa and MCF-7 cells were analyzed for endogenous STK16 using the anti-STK16 antibody. (b) HeLa, NIH-3T3 and MCF-7 cells were fixed and stained for endogenous STK16 by the anti-STK16 antibody. (c) HeLa and MCF-7 cells stably transfected with STK16-GFP-FLAG-WT were harvested for immunofluorescence using the anti-STK16 and anti-GFP antibodies. Scale bar: 10 μm.

Figure S2

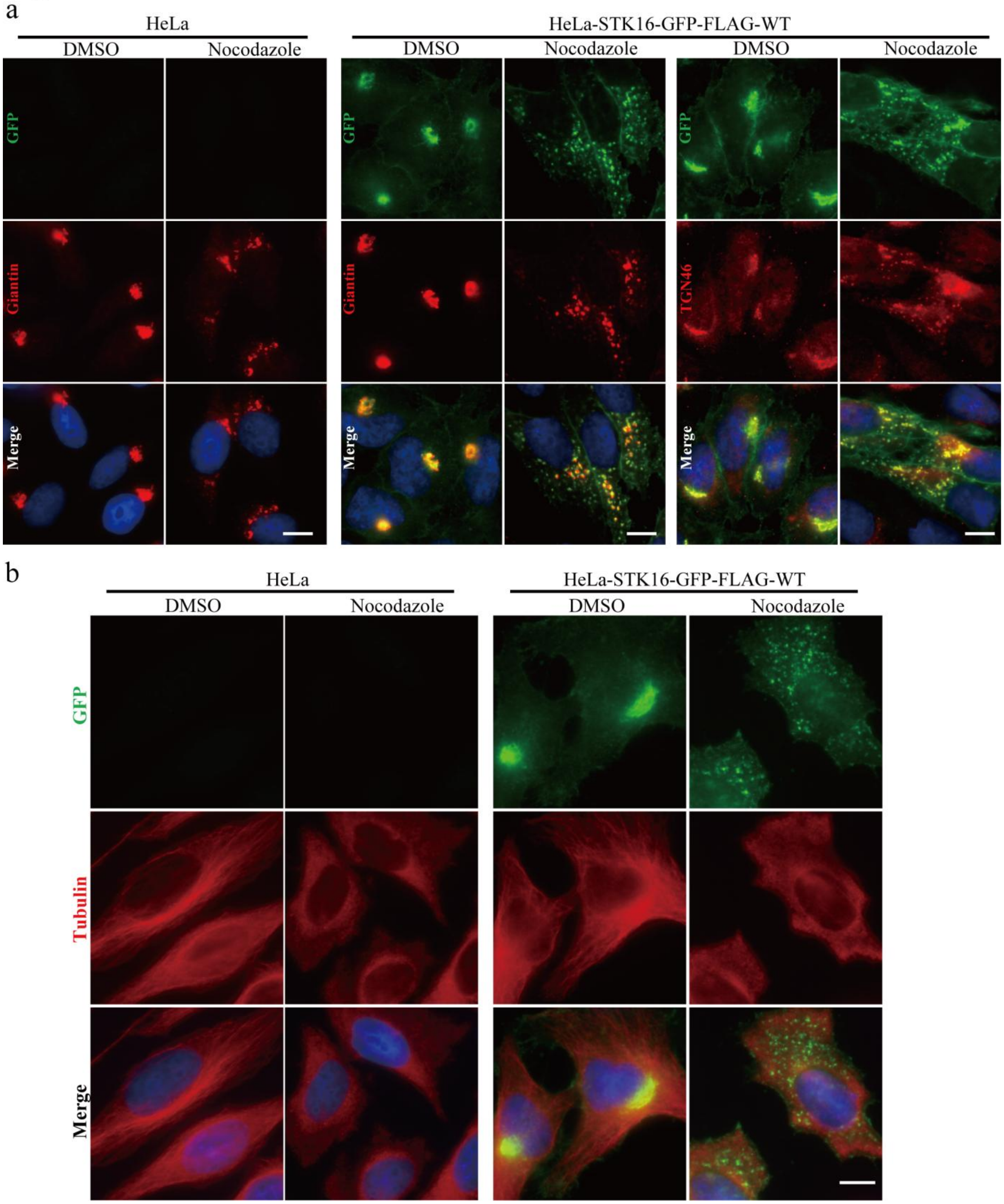

Figure S2. STK16 colocalizes with Golgi and TGN markers in Nocodazole-treated cells. HeLa and HeLa-STK16-GFP-FLAG-WT cells were treated with 10 μg/mL Nocodazole for 2 hours, and then fixed and stained for GFP and Giantin/TGN46 in (a), or GFP and tubulin in (b). Representative images are shown. Scale bar: 10 μm.

Figure S3

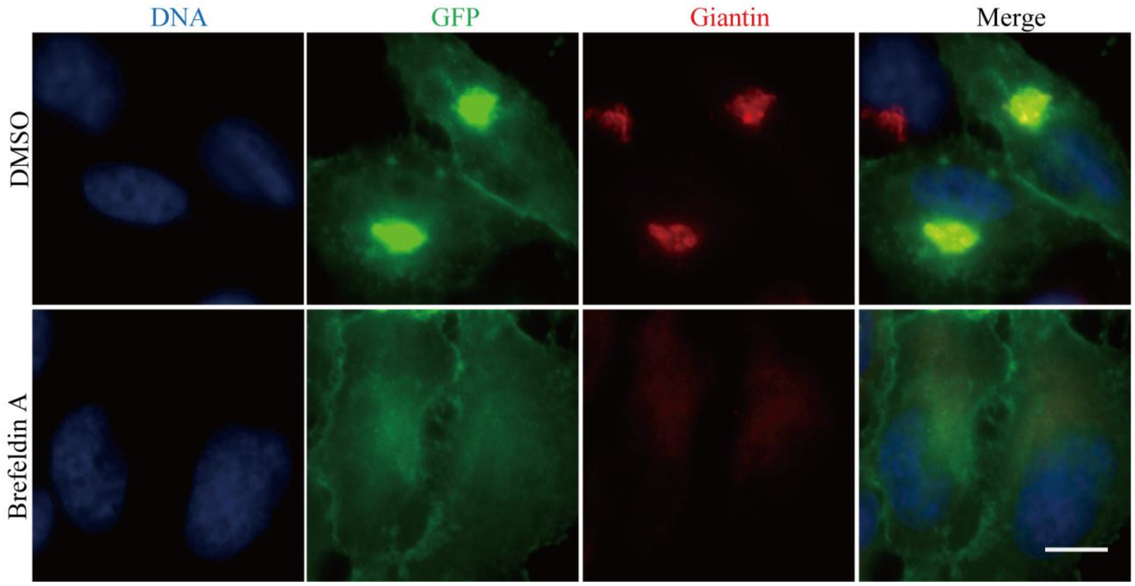

Figure S3. The Golgi localization of STK16 was dispersed by Brefeldin A treatment. HeLa-STK16-GFP-FLAG-WT cells were treated with 10 μg/mL Brefeldin A for 2 hours, fixed and stained for GFP and Giantin. Scale bar: 10 μm.

Figure S4

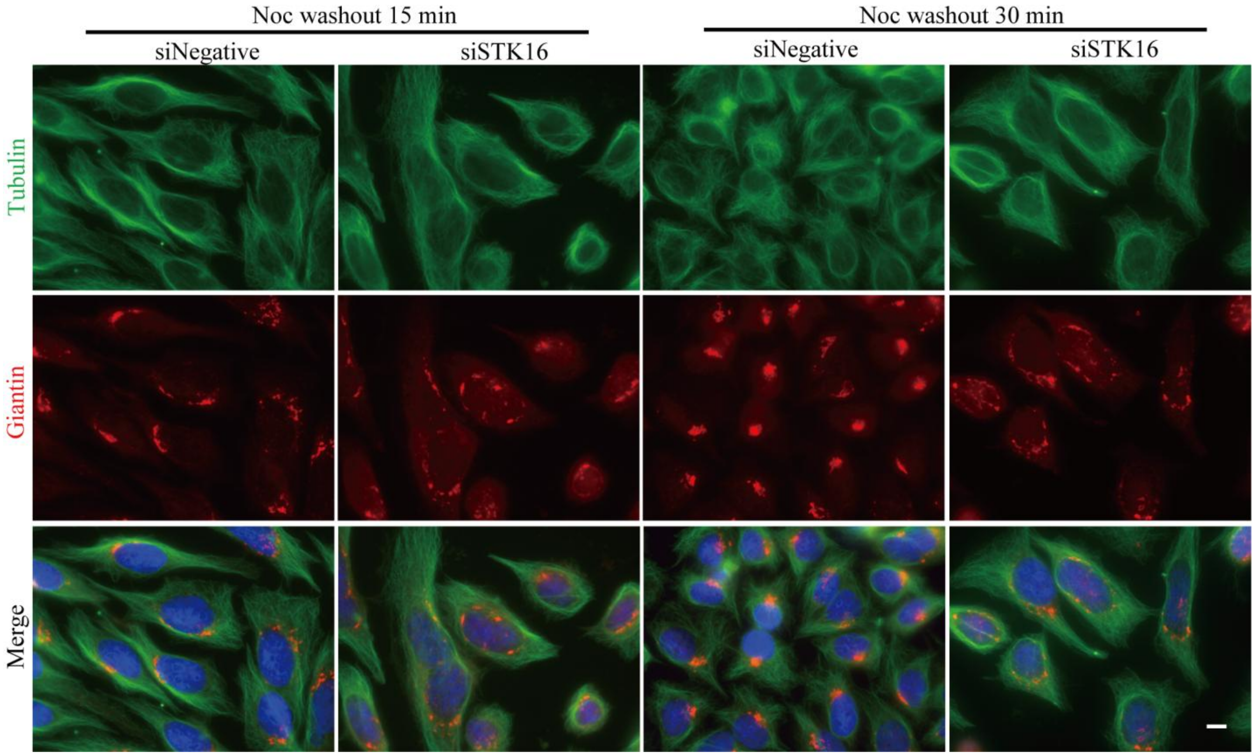

Figure S4. STK16 depletion impairs Golgi assembly. HeLa cells transfected with control or STK16 siRNAs for 72 hours were incubated with 10 µg/mL Nocodazole for 2 hours. After Nocodazole washout for 0, 5, 15, 30, 60, 120 minutes, cells were fixed and stained for tubulin (green) and Giantin (red). 15 and 30 minutes results are shown. Representative results are show. Scale bar: 10 µm.

Figure S5

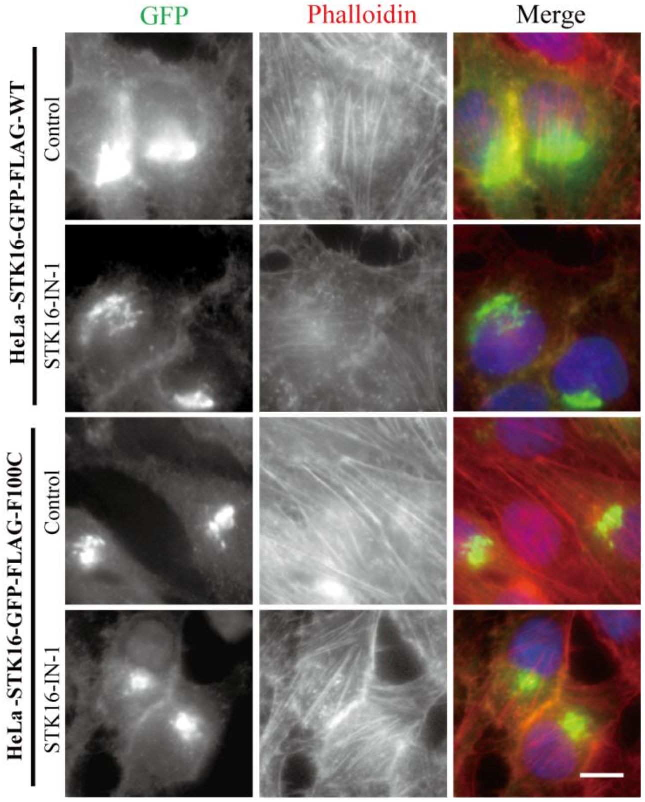

Figure S5. HeLa-STK16-GFP-FLAG-F100C rescues the actin cytoskeleton alteration caused by STK16-IN-1. HeLa-STK16-GFP-FLAG-WT and HeLa-STK16-GFP-FLAG-F100C cell lines were treated with 10 μM STK16-IN-1 for 6 hours. Cells were fixed and stained with anti-GFP (green) and fluorescently labeled Phalloidin (red). Representative results are show. Scale bar: 10 μm.

Figure S6

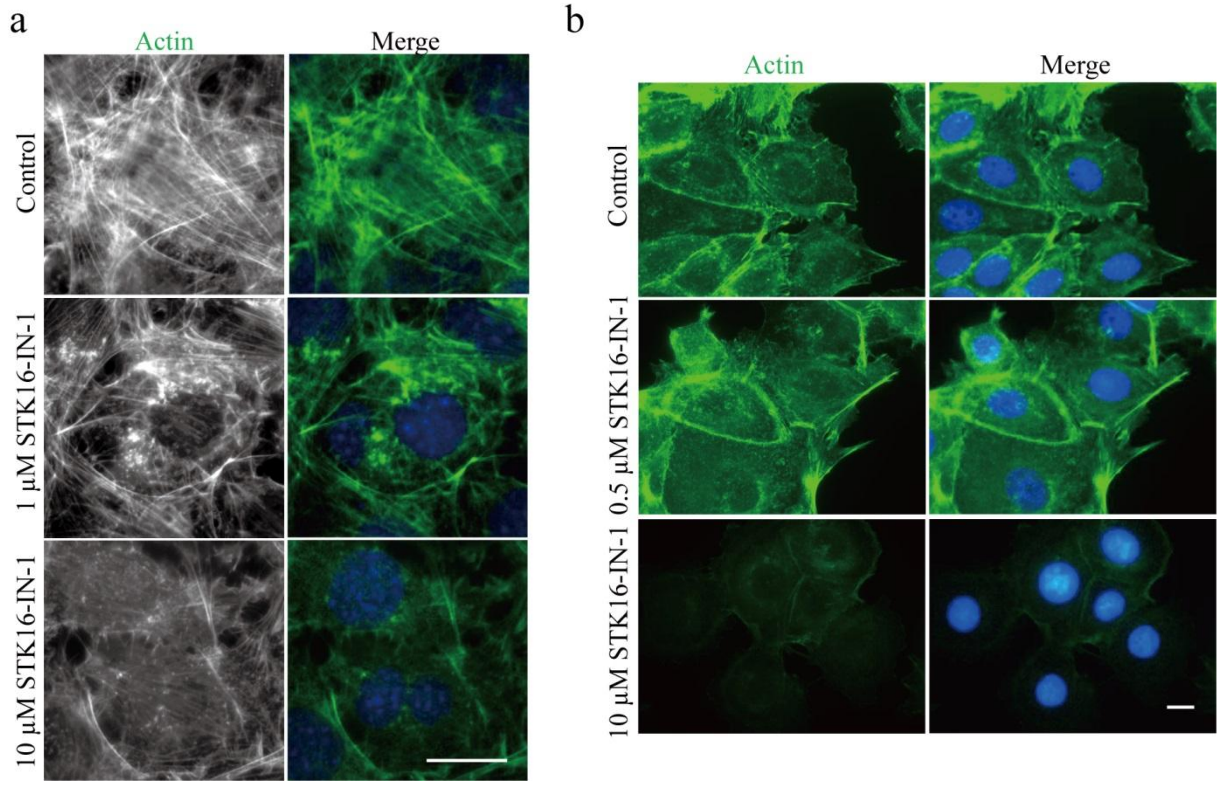

Figure S6. Actin cytoskeleton is decreased significantly by STK16-IN-1 in both NIH-3T3 and MCF-7 cells. Mouse embryonic fibroblast NIH-3T3 (a) and human breast cancer MCF-7 (b) cells were plated and incubated with DMSO, or different concentrations of STK16-IN-1 for 72 hours before they were fixed and stained with fluorescently labeled Phalloidin (green) and DAPI (blue). Representative images are show. Scale bar: 10 μm.

Figure S7

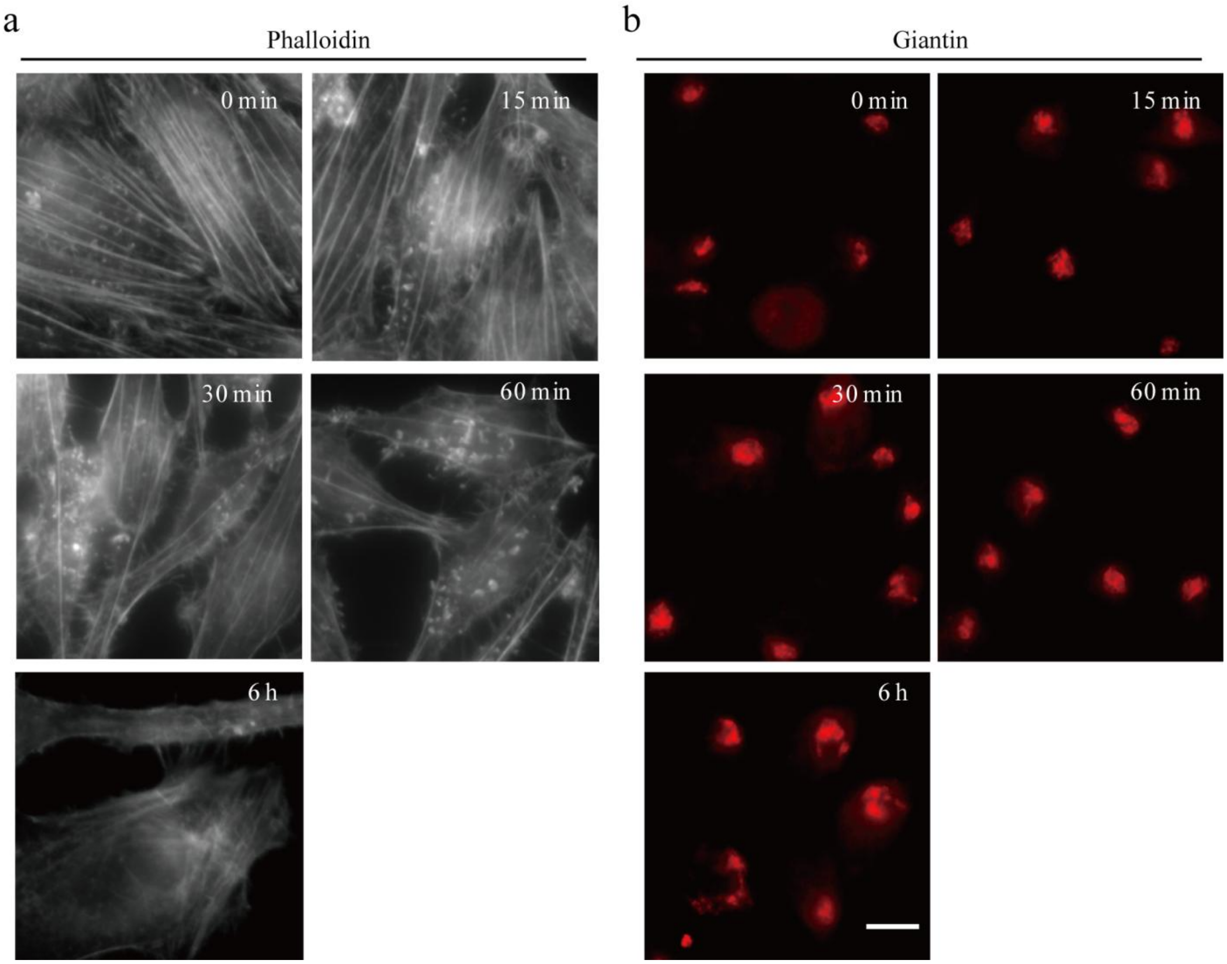

Figure S7. STK16-IN-1 disrupts actin stress fiber earlier than Golgi complex. HeLa cells were treated with 1 μM STK16-IN-1 for 0, 15, 30, 60 minutes or 6 hours before they were harvested for immunofluorescence using fluorescence-labeled phalloidin (a) or anti-Giantin antibody (b). Phalloidin and anti-Giantin were stained separately here. Experiments were repeated at least three times and representative results are shown. Scale bar: 10 μm.

Figure S8

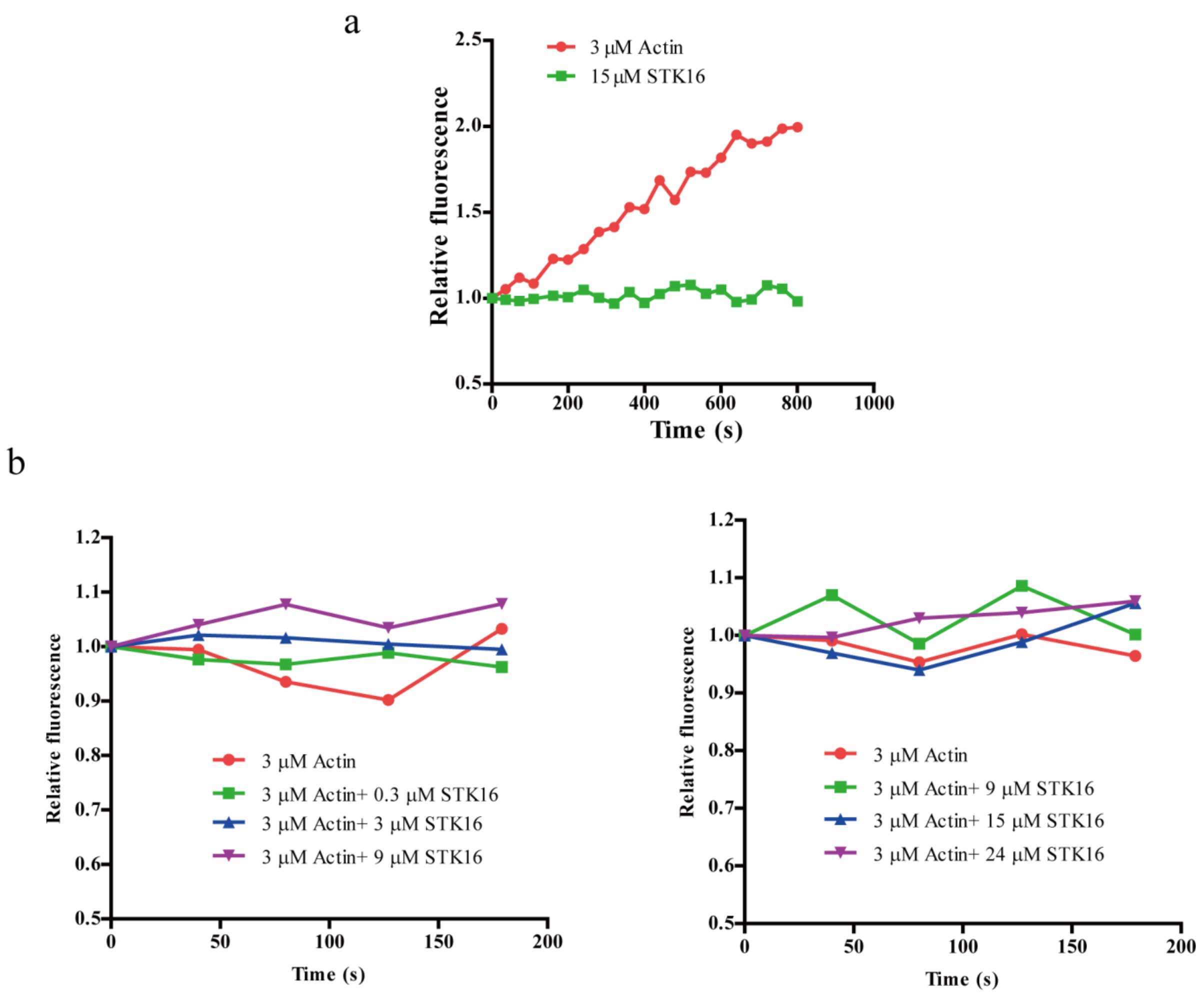

Figure S8. Fluorescence of pyrene-actin and STK16. (a) The graph shows linear increase in F-actin as a function of time as measured by the relative fluorescence signal emitted by 3 µM rabbit skeletal muscle actin mixed with 10% pyrene-actin. 15 µM STK16 has no fluorescence signal and it does not change over time. (b) The graph shows no fluorescence increase without polymerization buffer. 3 µM rabbit skeletal muscle actin mixed with 10% pyrene-actin were incubated with different concentrations of STK16 protein and the samples were read for 3 minutes to establish a baseline fluorescent measurement.

Figure S9

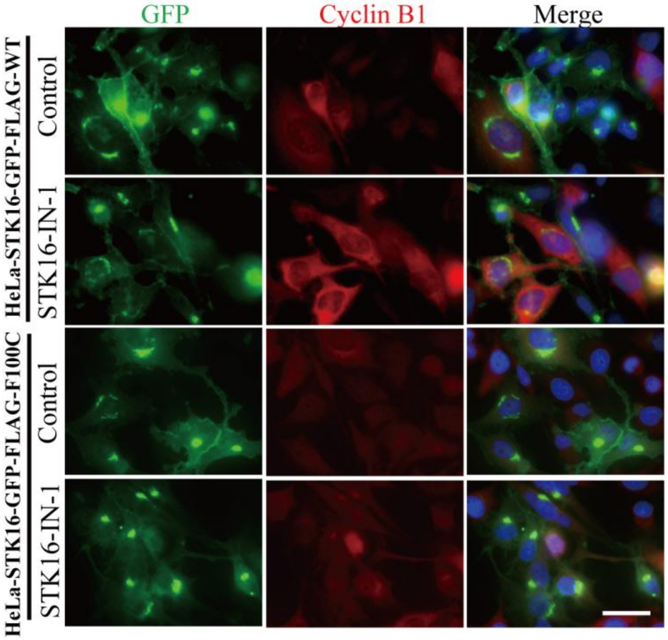

Figure S9. F100C mutant rescues the G2 phase arrest caused by STK16-IN-1. HeLa-STK16-GFP-FLAG-WT and HeLa-STK16-GFP-FLAG-F100C cell lines were treated with 10 µM STK16-IN-1 for 6 hours before they were fixed and stained with anti-GFP (green) and anti-Cyclin B1 (red). Representative results are show. Scale bar: 10 µm.

Figure S10

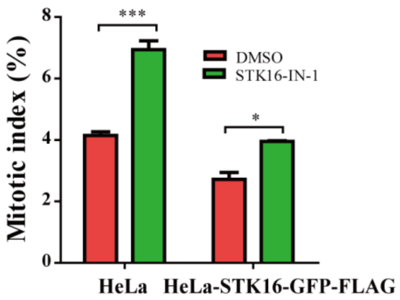

Figure S10. The kinase activity of STK16 is critical for its regulation of cell cycle. HeLa and HeLa-STK16-GFP-FLAG cell lines were treated with 10 µM STK16-IN-1 for 6 hours, and then fixed for immunofluorescence using anti-pH3 antibody and DAPI. Quantifications of percentage of mitotic cells were shown. The data were analyzed using a student's *t*- test (*p < 0.05, ***p < 0.005). Data show mean ± SEM, n = 3.
